# Synthesis and characterization of miniaturized aptamer-based monolithic sorbent for selective extraction of β-amyloid peptides from cerebrospinal fluid

**DOI:** 10.1007/s00216-025-06085-7

**Published:** 2025-09-01

**Authors:** Israel Donizeti de Souza, Caroline Fernandes Grecco, Maria Eugênia Costa Queiroz, Valerie Pichon, Audrey Combès

**Affiliations:** 1 Department of Analytical, Bioanalytical Sciences and Miniaturization (LSABM), Chemistry, Biology and Innovation (CBI), UMR 8231 ESPCI Paris - CNRS, ESPCI Paris, PSL University, 10 Rue Vauquelin, 75005 Paris, France; 2 Departamento de Química da Faculdade de Filosofia, Ciências E Letras de Ribeirão Preto (DQ-FFCLRP), Universidade de São Paulo, Ribeirão Preto, São Paulo Brazil

**Keywords:** Amyloid beta peptide, Aptamers, Biomarkers, Cerebrospinal fluid, Miniaturized oligosorbents, Monolith

## Abstract

The ratio between beta-amyloid (Aβ) peptides 40 and 42 is recognized as a biomarker for Alzheimer's disease, playing a significant role in early diagnosis and disease progression monitoring. Aβ peptides are present at trace levels in cerebrospinal fluid, therefore, developing a new selective extraction procedure is essential for isolating targeted biomarkers from the matrix interferents, ensuring accurate identification and quantification. In this study, a hybrid organic-silica monolith was synthesized in a 530 µm inner diameter-capillary and used for the covalent grafting of beta amyloid peptide aptamers. The resulting miniaturized oligosorbent (mOS) was applied to selectively extract Aβ peptides 40 and 42 from artificial cerebrospinal fluid (CSF) samples. The immobilization procedure achieved grafting yields higher than 90% leading to a dense coverage of Aβ aptamers (655 + 15 pmol.µL^−1^ of oligosorbent, n = 3, RSD = 2.3), and in a capacity exceeding 50 pmol.µL^−1^ of mOS. After optimization in pure media, an extraction recovery of 74% and 31% for Aβ40 and Aβ42 peptides, respectively was reached on this mOS. The developed method including extraction on mOS and LC–MS analysis achieved LLOQ values of 0.1 ng.mL^−1^, precision and accuracy with CV and RSD values ranging from 1.0% to 12.9% and −4.7% to 11.1%, respectively. This method was successfully applied to selectively extract Aβ peptides from artificial CSF samples, effectively isolating the two Aβ targeted peptides from this complex biological fluid and enhancing trace-level analysis reliability.

**Graphical Abstract:**

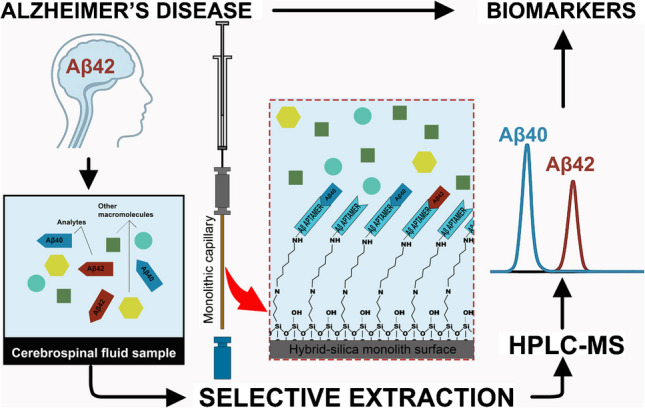

**Supplementary Information:**

The online version contains supplementary material available at 10.1007/s00216-025-06085-7.

## Introduction

Alzheimer's disease (AD) is one of the most common types of neurodegenerative disorders. The prevalence is 46.8 million people affected by AD worldwide according to World Alzheimer´s Report [1]. There are regional differences in the prevalence among individuals over 60, with Southeast Asia reporting a prevalence of 2.9%, Europe of 6.5%, and other regions reporting rates between 3.1% and 5.7% [2]. AD symptoms include memory, other cognitive impairments, and dementia in the last stage of the disease. However, it has been documented that AD begins as an asymptomatic biological process including substantial accumulation of amyloid-β (Aβ) plaques and neurofibrillary tangles within their brains, accompanied by a cascade of pathological processes such as neuroinflammation [2]. When amyloid precursor protein, mainly present in the brain is enzymatically cleaved, it can produce different amyloid peptides like Aβ40 (DAEFRHDSGYEVHHQKLVFFAEDVGSNKGAIIGLMVGGVV) or Aβ42 (DAEFRHDSGYEVHHQKLVFFAEDVGSNKGAIIGLMVGGVVIA) which is 2 amino acids longer and also has a greater tendency to aggregate. The use of amyloid positron emission tomography (PET) imaging technique applied to brain samples and the immunoassays assays quantification methods applied in cerebrospinal fluid samples have helped in understanding the metabolism of this disease at the cellular level [3]. The Aβ42/Aβ40 ratio in CSF are well-consolidated biomarker for AD diagnosis and prognosis [4].

Quantitative methods based on reverse phase liquid chromatography coupled with tandem mass spectrometry (RPLC-MS/MS) can overcome some limitations associated with immunoassays assays for example the use of highly specific antibodies and reagents, short linearity ranges, cross-reactivity among peptides, batch-to-batch variability, and their high sensitivity to matrix interferents [5]. Reference methods for determining Aβ peptides in CSF samples use micro-elution solid phase (SPE) 96-well plate format as an enrichment step (sample preparation) before RPLC-MS/MS analysis [6, 7]. This method allowed the analysis of intact Aβ peptides. However, this method presents low selectivity and the sample preparation step is difficult to online coupling with RPLC-MS/MS system. More recently, a new stationary phase based on zwitterionic polymeric ionic liquid for *fiber-in-tube* SPME devices has been developed [8]. Despite the good extraction efficiency of this innovative extraction sorbent, the preparation of the *fiber-in-tube* SPME extraction capillary is a laborious process because the packaging of the fibers in the capillary is done manually, which makes it difficult to obtain adequate reproducibility in the synthesis. Therefore, the development of new miniaturized methods and especially the development of reproducible and more selective sorbents to selectively extract Aβ peptides from biological fluids are mandatory.

Selective sorbent can be obtained by the immobilization of aptamers on a solid phase. Aptamers are single-stranded DNA or RNA oligonucleotides selected from a large library using SELEX procedure. They exhibit high affinity and specificity for specific targets, ranging from individual molecules to entire organisms [9]. They can adopt complex three-dimensional structures, allowing them to bind targeted molecules through interactions like electrostatic forces and hydrogen bonding [9]. Compared to antibodies and molecularly imprinted polymers, they present advantages including high stability, non-toxicity, affordability, straightforward synthesis and tailorable chemical modification, and absence of immunogenicity. Moreover, aptamers can be regenerated faster than antibodies [10]. These advantages enable their use in various areas, including protein purification, enantiomer recognition, drug development, sensors, and sample preparation techniques [11].

Aptamer-functionalized affinity sorbents have been evaluated with different microextraction techniques. For example, aptamer-functionalized affinity hybrid organic-silica monolithic capillaries have been valuable prepared for the selective extraction of α-thrombin from human serum [12, 13], bisphenol A from milk and serum samples [14], and ochratoxin A from beer [15, 16] and wine [15]. These affinity sorbents have provided high selective extraction of target analytes and increased the efficiency of sample clean-up. The aptamer-functionalized affinity monolithic capillary columns combine the selectivity of aptamers with large column loading capacity and high mass transfer rate of monolithic material [17].

To date, some studies have reported aptamers specific for Aβ peptides and applied them as biosensing [18]. However, none of that had explored these aptamers in microextraction techniques associated with RPLC-MS analysis. The purpose of this study was thus to highlight the potential of aptamer-functionalized affinity hybrid organic-silica monolithic capillaries. First, the screening of the synthesis conditions of homogeneous hybrid organic-silica monolithic capillary is presented. Then, this monolithic sorbent was used as support for the covalent immobilization of amyloid peptide Aβ aptamers. Also, characterization, optimization of the main extraction parameters, and figures of merits of analytical validation are presented in this study.

## Experimental

### Reagents and analytical standards

The analytical standards, Aβ synthetic peptides (Aβ38, Aβ40, and Aβ42), and the internal standards, nitrogen-15 stable-isotope labelled amyloid beta peptides (^15^N_53_-Aβ40 and ^15^N_55_-Aβ42), were all acquired from rPeptide (Athens, USA). The 5′-amino-modified C12-aptamers specific for Aβ were synthesized and purified by HPLC by Eurogentec (Angers, France). The sequence of these aptamers was 5′-GCCTGTGGTGTTGGGGCGGGTGCG-3′ (apt1) [19] and 5′-GCCTGTGTTGGGGCGGGTGCG-3′ (apt1) [20]. The binding buffer (BB) was composed by Tris–HCl buffer solution (10 mM Tris–HCl, 150 mM NaCl, 5 mM KCl, pH 7.4). Aminopropyltriethoxysilane (APTES), tetraethyl orthosilicate (TEOS), hexadecyltrimethylammonium bromide (CTAB), glutaraldehyde, sodium chloride, K_2_HPO_4_, Tris–HCl, and NaCNBH_3_ were from Sigma-Aldrich (Saint-Quentin Fallavier, France). Potassium dihydrogen phosphate (KH_2_PO_4_) and hydrochloric acid were from VWR (Fontenay-sous-bois, France). Sodium hydroxide (NaOH) was from Merck (Darmstadt, Germany). HPLC-grade acetonitrile, HPLC-grade methanol, and anhydrous ethanol (EtOH) solvents were from Carlo Erba (Val de Reuil, France). Fused-silica capillaries (530 μm i.d.) were from Polymicro (Photon Lines, St. Germain en Laye, France). Polypropylene Protein LoBind tubes were purchased from Eppendorf (Hamburg, Germany). High-purity water was obtained using a Milli-Q purification system (Millipore, Saint-Quentin en Yvelines, France). Artificial CSF was composed of 122 mM NaCl, 3 mM KCl, 0.4 mM KH_2_PO_4_, 1.3 mM CaCl_2_, 25 mM NaHCO_3_, 1.2 mM MgSO_4_, and 0.4% bovine serum albumin [21].

### Chromatographic and mass spectrometry conditions

The analyses were carried out with an Agilent 1200 series (Agilent Technology, Massy, France) LC system coupled to the Ultivo Triple Quadrupole mass spectrometer equipped with electrospray ionization source operating in positive mode. Samples were injected by Low Flow HiP autosampler, injection volume was set at 8 µL. The peptides [Aβ38 (*m/z* 1033.8), Aβ40 (*m/z* 1096.2), and Aβ42 (*m/z* 1129.5)] were analyzed in single ion monitoring mode (dwell time of 100 ms and fragmentor of 130 V and 150 V for Aβ40 and Aβ42 respectively). Other MS operational parameters were capillary voltage, 5.00 kV; desolvation temperature, 350 °C; and desolvation gas flow, 10 L.min^−1^ (N_2_, 99.9% purity); nebulizer, 15 psi.

An XSelect®Peptide CSH C18 (2.1 × 150 mm, 3.5 µm, 130 Å) chromatographic column was used. The mobile phase consisted of water (A) and acetonitrile (B) containing 0.3% ammonium hydroxide. The elution gradient started with 10% B and increased to 20% B in 2 min followed by an increase from 20 to 25% B from 2 to 5 min, and from 25 to 70% B from 5 to 12 min. Then the mobile phase returns to 10% B in 1 min. Finaly, the mobile phase was kept in 10% B for 4 min to allow the system to equilibrate for the next injection. The mobile phase was pumped at 150 µL.min^−1^ and the chromatographic column was maintained at 30 ˚C.

### Preparation of aptamer-modified monolithic sorbents

#### Hybrid organic-silica monolithic sorbent

A 50 cm segment of fused silica capillary (530 μm i.d.) was first activated at 15 μL.min^−1^ with a 1 M sodium hydroxide solution during 60 min. The capillary was then flushed at the same flow and for the same time successively with water, 0.1 M HCl, water, and methanol before flushing with nitrogen and drying in a GC oven for 5 h at 160 °C. The monolithic organic silica capillaries were synthesized *in-situ* using sol–gel approach. Different proportions of TEOS, APTES, water, CTAB, and methanol (as detailed in Table 1) were mixed in order to form a homogeneous solution. Subsequently, 15 cm segments of the activated capillaries were filled with the polymerization mixture, sealed at both ends with silicone rubber, and heated at 40 °C for 48 h. Finally, the capillary was rinsed with methanol to flush out the remaining reagents.

**Table 1:** Synthesis mixture composition and measured permeability

Capillary	TEOS (mmol, µL)	APTES (mmol, µL)	Monomers ratio	WATER (µL)	CTAB (mg)	EtOH (µL)	Permeability (× 10^–13^ m^2^)
CapS1	0.50, 112	0.50, 118	1:1	32	8	215	1.95
CapS2	0.50, 112	0.25, 59	2:1	32	8	274	4.26
CapS3	0.25, 56	0.50, 118	1:2	32	8	271	4.56; 6.33
CapS4	0.50, 112	0.50, 118	1:1	32	4	215	1.78; 2.16
CapS5	0.50, 112	0.50, 118	1:1	32	12	215	3.37; 1.22

TEOS: tetraethoxysilane, APTES: (3-aminopropyl)-triethoxysilane; CTAB: Cetyltrimethylammonium bromide; EtOH: ethanol

#### Aptamer grafting onto hybrid organic-silica monolithic sorbent

The volume and the flow-rate of the solutions percolated through the 530 µm id capillary during the immobilization procedure have been adapted from a previous study [16] using monolith synthesized in 100 µm diameter capillaries in order to keep constant the ratio between the volume of solution used for each step and the dead volume of the capillary and the linear velocity [16]. The monolithic sorbent was thus first activated with glutaraldehyde (10%, v/v), dissolved in 100 mM phosphate buffer (pH 8.0), for 16 h at 8.4 µL.min^−1^. Forty minutes before the end of this step, grafting solution containing aptamer at 1 mg.mL^−1^ in phosphate buffer solution 100 mM, pH 7.4, was heated at 90 °C for 3 min, followed by cooling at room temperature for 30 min. The grafting solution was then percolated through the monolithic capillary during 25 h at 80 nL.min^−1^. To remove the unbound aptamers, cleaning solution composed of phosphate solution buffer (100 mM, pH 8.0) was percolated through the capillary for 30 min at 5.6 µL.min^−1^. The resulting miniaturized mOS, named respectively mOS1 and mOS2 according to the aptamers grafted, were rinsed during 3 h at 4 µL.min^−1^ with BB solution containing NaCNBH_3_ at 5 mg.mL^−1^. This step was crucial for deactivation of non-reacted glutaraldehyde groups and stabilization of the ligation between the NH_2_ groups of aptamers and glutaraldehyde [13]. Each mOS was washed with Tris–HCl buffer solution for 3 h at 4 µL.min^−1^. Finally, it was washed with water/methanol 70:30 (v/v) mixture for 30 min at 14 µL.min^−1^. All the solutions used during the grafting procedure for both aptamers were collected for the determination of the grafting yields (see item S1 and Fig S1 in supplementary information (SI) for a detailed quantification procedure of the aptamers). The mOS capillaries were cut to a final 10 cm length (approximately 2.5 cm at each extremity were removed) and stored at 4 °C in BB until further characterization.

### Characterization

#### Permeability assay

The permeability [16] of the monolithic sorbent was be calculated using the Darcy’s law which expresses the permeability as a function of the applied flow rate of a solution:

$$K=\frac{4\times \eta \times L}{\pi \times {\left({d}_{i}\right)}^{2}\times \Delta P}\times F$$

*K* is the permeability (m^2^), *F* is the applied flow rate (m^3^.s^−1^), η is the viscosity of the percolated solvent (methanol, η = 0.54 × 10^–3^ Pa.s), L the length of the capillary (m), ∆P the measured backpressure (Pa), and finally *d*_*i*_ is the capillary internal diameter (m). To perform the permeability test, one end of the hybrid organic-silica monolithic capillary (100 mm × 530 μm) was connected to a nano HPLC pump Agilent 1200 series (Agilent Technology, Massy, France). The back pressure of the system was then measured by pumping methanol at different flow rates ranging from 20 to 200 µL.min^−1^.

#### Scanning electron microscopy (SEM)

The mOS was cut in cross-sectional position with a column cutter and placed in the SEM holder. No metallization was needed. SEM images were obtained with a Hitachi S-3600N scanning electron microscope operated with a beam energy (from 5 to 20 keV).

### Optimization of the extraction procedure in *off-line* mode

The capillary was connected to 250 µL-syringe gas tight (see Fig S2 in SI). In this setup, a syringe pump was used to percolate sample (6 µL.min^−1^), washing (6 µL.min^−1^), and elution solutions (12 µL.min^−1^) through the mOS. Extraction procedure in *off-line* mode was carried out in an air-conditioned laboratory at 23 °C. First, 60 µL of BB spiked with the targeted Aβ peptides at 1 µg.mL^−1^ were percolated. The washing step was optimized by testing a first step of washing with different volumes of BB (48–120 µL) followed by the adding of a second washing step with pure water whose volume (21–63 µL) was optimized to eliminate ions that affect LC–MS analysis. The volume (48–150 µL) of the elution step which was carried out with a mixture water/acetonitrile 70:30 *v/v* was finally optimized. The elution solution was dried under nitrogen stream and the dry residue was resuspended with a mixture water/acetonitrile 90:10, *v/v* containing 0.3% of NH_4_OH prior to LC–MS analysis.

### Maximum sorption capacity

Using the optimized extraction conditions, 60 µL of BB spiked with increasing quantity of Aβ peptides (concentration range from 0 to 180 µg.mL^−1^ were percolated through the extraction capillary. The amount of Aβ peptides extracted from the sorbent was plotted as a function of the Aβ amount percolated.

### Figure of merits of the developed methods

Calibration curves were generated by linear regression of the ratio between the targeted Aβ peptides and the internal standard (Y) peak areas *versus* the Aβ peptide concentrations (X, ng.mL^−1^) in synthetic CSF. The lower limit of quantitation, LLOQ, corresponded to the lowest concentration on the analytical curve that could be quantitatively measured with acceptable precision and accuracy (within 20%).

Accuracy (relative standard deviation, RSD), precision (coefficient of variation, CV), and extraction recovery were determined by replicate the whole analyses (n = 3) including the extraction on the mOS, the evaporation of the elution fraction, the resuspension and the LC–MS analysis of the artificial CSF spiked with the Aβ 40 and 42 peptides at 4 different levels of concentration (low, medium, high, and upper limit of quantification). The low, medium, high, and upper limit of quantification concentrations were set at 0.6, 2.5, 8.0, and 13.0 ng.mL^−1^, respectively. These values were selected according to the expected level of Aβ peptides in real CSF samples [22, 23].

Carry-over was assessed by injecting aliquots of the same non-spiked sample after analysis of the sample spiked with the analytes at the concentration corresponding to the upper limit of quantification (ULOQ). Carry-over in the non-spiked sample was evaluated immediately after the ULOQ sample should not be greater than 20% of the analyte signals in the LLOQ chromatogram, and not greater than 5% of the IS.

Reusability and long-term stability of the capillary were evaluated based on extraction efficiency over multiple extractions both in pure and in complex media performed on a period of 4 months.

## Results and discussion

### Synthesis of the hybrid organic-silica monolithic

**Fig. 1:**
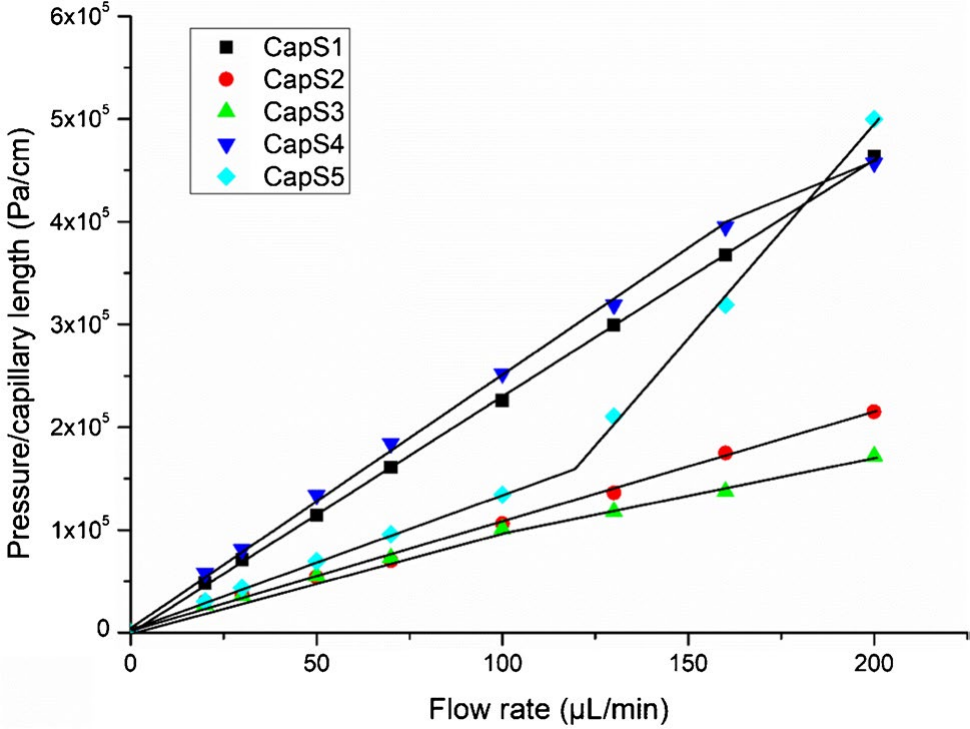
Evolution of back-pressure-flow rate generated when percolating methanol through the synthesized hybrid-silica monolithic capillaries (100 mm × 530 μm i.d., see synthesis conditions depicted in Table 1) and measured at 20 ºC

The hybrid organic-silica monolith was in situ synthesized by the sol–gel process consisting of the hydrolysis and co-condensation of two precursors, TEOS and APTES, in the presence of a structure-directing agent, CTAB, that allows the formation of large-through pores. Hybrid organic-silica monolithic stationary phase have been reported successfully in low internal diameter fused-silica capillary (< 200 µm) [16, 24]. In this study, the monolithic sorbent was prepared in a 530 µm (i.d.) fused-silica capillary. The challenge of using a higher diameter capillary was to achieve a homogeneously monolithic phase properly attached to the internal surface of the capillary and with a permeability compatible with the percolation of solutions of the SPE procedure. For this purpose, different conditions of synthesis (Table 1) were evaluated thanks to the measure of the permeability (K), which reflects through-pore size and external porosity. In these experiments, the volume of ethanol was adjusted to keep the same the final volume of all the polymerization solution. Figure 1 shows the back pressure generated by the different monoliths synthesized according to the flow rate. Keeping the TEOS/APTES molar ratio at 1:1 and the same volume of solution, the effect of different CTAB concentrations in polymerization mixture, that strongly influenced the mechanical stability of the monolith, was evaluated (CapS1, CapS4 and CapS5). Increasing CTAB concentration, led to increase the permeability of the monolith (as shown in Fig. 1). Indeed, for the capillaries CapS4 and CapS5, which correspond to the lower and higher concentration of CTAB, respectively, it is possible to see negative and positive breaks in the permeability curve, respectively. These breaks resulted from the cracks in the monolith structure at a high flow rate. CapS3 also present a negative break in the permeability curve. Probably, the higher amount of APTES increases the alkalinity of the reaction media, which prejudicated the formation of the tridimensional silica network [25]. The lower CTAB concentration (capillary CapS4) tends to result in the formation of a less permeable and rigid structure. The higher back pressure of about 3.8 × 10^6^ Pa caused cracks in the monolith structure and reduced the slope of the permeability curve. A higher amount of CTAB also produces a monolith with lower mechanical stability (capillary S5). This monolith could be used at a back pressure of less than 1.5 × 10^6^ Pa. These results were in accordance with literature showing that the porous structure of the monolith is directly proportional to the molar CTAB concentration [26]. Keeping this molar ratio of CTAB (8 mg added) constant in the polymerization mixture and playing on the molar ratios between TEOS and APTES (CapS2 and S3) leads to monoliths with much greater porosity.

**Fig. 2:**
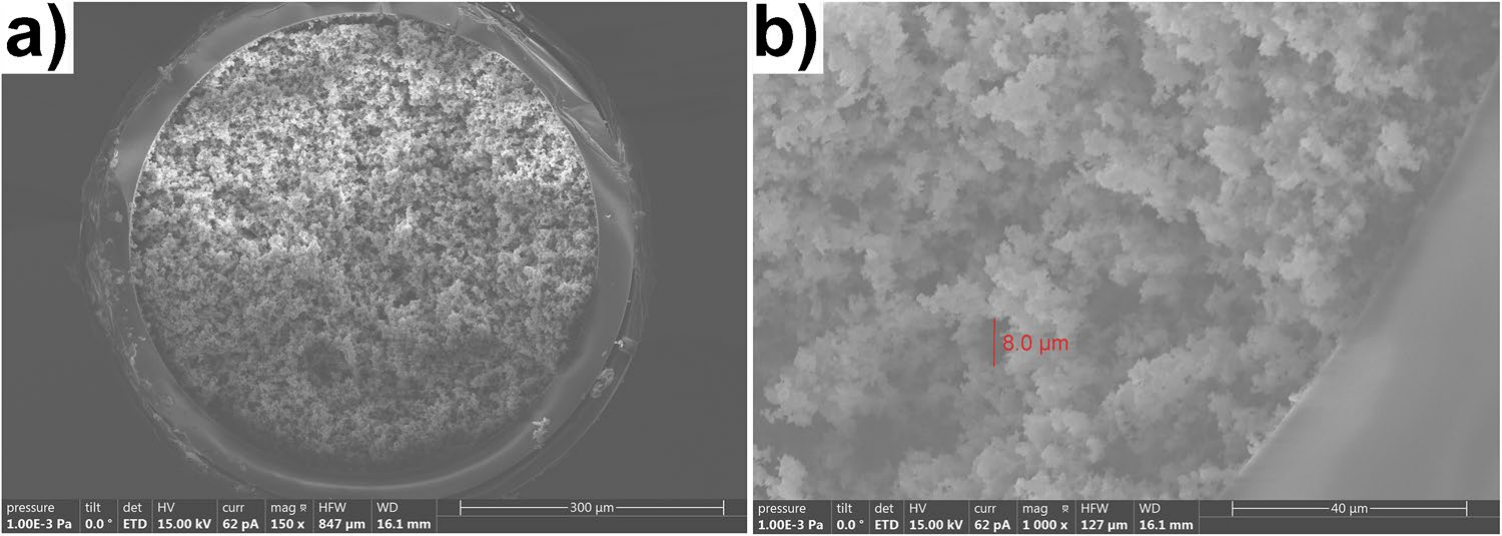
SEM images of CapS1 containing the hybrid silica monolith sorbent with different amplifications, 150x (**a**) and 1000x (**b**); the arrow in the figure shows the approximate size of a macropore

Although CapS2 presented stable permeability, the structure of the monolith was not homogeneous. Failures in the monolith structure were observed after percolation of aqueous solution for 16 h (see Fig S3 in SI). Altogether, only the capillary CapS1, showed good mechanical stability in the flow rate range examined. The permeability of CapS1 was 1.95 × 10^–13^ m^2^ (RSD = 13%, n = 3). This value was very close to those reported by Brothier et al., 6.15 × 10^−14^ m^2^ who synthesized a monolith under the same conditions but in a 100 µm i.d. capillary [16]. Instead CTAB, Xu et al. applied poly (ethylene glycol) and urea, as porogen, to prepare monolithic capillary with tetramethyl orthosilicate (TMOS) and 3-(trimethoxysilyl)-propylmethacrylate (γ-MAPS) precursors. The authors obtained an hybrid organic-silica monolithic capillary (100 μm I.D.) with permeability of 0.82 × 10^−14^ m^2^ [27]. Compared to this study, the use of CTAB ensured capillaries with better permeability without compromising the physical stability of the monolithic structure. As shown in Fig. 2, this monolithic structure presents a homogeneous porous structure tightly attached to the capillary inner wall. These properties guarantee effective mass transfer and high stability for the capillary. It also demonstrates the presence of microporous structure distributed around the monolith structure. Therefore, the CapS1 capillary was selected as monolithic support for grafting the Aβ aptamers for the rest of this study. Three new CapS1 were synthesized and the permeability was measured. The coefficient of variation of the permeability was lower than 10%, which indicate good reproducibility in the synthesis reproducibility. Then, these monolithic capillaries (CapS1) were used as support for grafting the different aptamers, apt1 and apt2.

### Preparation of the monolithic oligosorbent

Chemical synthesis of aptamers allows modifications at their both ends for effective immobilization on various supports, tailored to the bonding nature. The amino modification has been described as the most common immobilization approach. In this study, two different 5′–NH2–Aβ-aptamers modified with C12 spacer, described in literature for having affinity for peptide Aβ40 (Kd = 25 nmol.L^−1^) [19, 20, 28] were immobilized on the synthesized monolith that possess amino group at it surface while using glutaraldehyde. These syntheses led to two distinct mOSs respectively named mOS1 and mOS2 according to the apt 1 and apt 2 grafted on the monolith. The C12 spacer is important to maintain the binding properties of the aptamers when it is bounded on the monolith surface [11].

Using the optimized conditions for the monolith’s synthesis, and the grafting procedure adapted from a previous study of our group (see Sections "Experimental"-"Conclusion") [16], grafting yields of 92% and 89% were obtained for mOS1 and mOS2, respectively. These values are in agreement with grafting yields (range 83.3 to 96.4%) of 100 µm id mOS monolithic capillaries [16]. The aptamer coverage densities have thus been determined to be 655 ± 15 pmol.µL^−1^ of mOS1 (n = 3, RSD = 2.3%) and 686 ± 20 pmol.µL^−1^ of mOS2 (n = 3, RSD = 2.9%) for mOS1 and mOS2, respectively. Based on the volume of the capillary (10 cm length = 22 µL) and considering that 1 mol of aptamer can trap 1 mol of Aβ, the theoretical maximum extraction capacity of mOS1 and mOS2 were calculated as 62 and 65 µg, respectively. Once the success of aptamer immobilization has been confirmed, the extraction conditions were optimized to ensure high extraction recovery in pure media for the peptide Aβ40 for which these aptamers have been specifically selected in literature but also of Aβ 42 peptide [29], which is of interest for diagnosis of AD.

### Optimization of extraction procedure

Usually, as the pH and salt composition have a significant impact on the aptamer's three-dimensional structure, the buffer used during the SELEX procedure [19, 30] was also used as percolation media in the extraction procedure to promote interactions between aptamers and targeted peptides. According to the selection buffer described [19], the extraction buffer (named binding buffer, BB) consisted of 10 mM Tris–HCl, 150 mM NaCl, 5 mM KCl, pH 7.4. Therefore, to investigate their retention on the mOS, Aβ peptides were diluted in this BB to prepare the percolation solution.

**Fig. 3:**
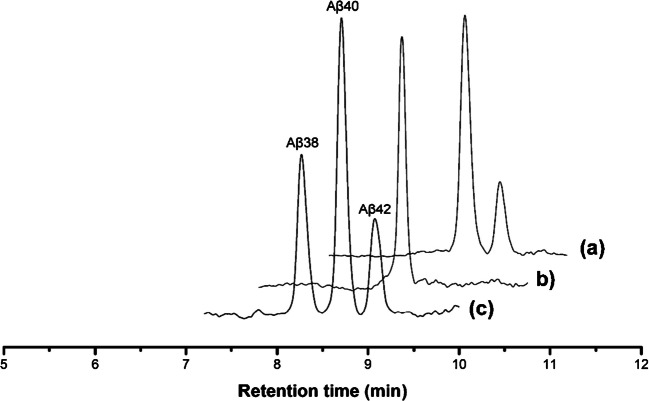
SIM chromatograms corresponding to the elution fraction issued of extraction procedure performed on CapS1 grafted with (**a**) mOS1, (**b**) mOS2, and (**c**) Aβ standard solution at 100 ng.mL.^−1^

It has been also demonstrated that’s crucial to keep the extraction flow rate constant to achieve good reproducibility in the extraction procedure [31]. Therefore, a simple experimental setup was built using a syringe pump (see Fig S2 in SI). To obtain maximum extraction performance of the mOS column, different conditions of washing: (i) composition of washing solutions (BB and water) and (ii) volume used during washing steps (21–63 µL corresponding to 1—3 dead volume -V_0_ of 21 µL- of the monolith) and volume (48–150 µL corresponding to 3—7 V_0_) of water/acetonitrile 70:30 *v/v* used as elution solution were evaluated. The optimization of the extraction procedure was carried out by percolating 60 µL of BB spiked with analytes at 1 µg mL^−1^, which corresponds to 0.1% of the theoretical maximum extraction capacity of mOS. Non-spiked BB was then used as washing solvent to remove potential interferents that may remain in the extraction capillary after percolation of samples. Then, the analytes retained in the capillary were eluted and diluted with acetonitrile containing ammonium hydroxide for injection into the HPLC–MS system. The addition of ammonium hydroxide solution was necessary for the adequate ionization of the analytes for the RPLC-MS/MS analysis. The preliminary results reported in Fig. 3 showed that the mOS1 was the only support able to completely trap during percolation the (74%) Aβ40 peptide and, in a lower extent (42%), Aβ42. This aptamer has been exploited to construct electrochemical sensors. In one published study, for example, an (Apt-1)-tethered gold nanoflower modified electrode for target capture and aptamer-tagged gold nanoparticles/Cu-metal organic framework conjugates were fabricated and combined as sandwich electrochemical aptasensor [30]. In another study, a biosensor was obtained by Apt-1 immobilized onto an Au electrode and incubed with Aβ peptide and graphite-like carbon nitride composite [32]. Both sensors reported the ability of Apt-1 to bind to Aβ40 and Aβ42 peptides.

It was intriguing to observe that mOS1 exhibited significantly higher selectivity for Aβ40 compared to Aβ42. Although these isoforms differ by only two residues at the C-terminus, such a minor variation can impart distinct structural characteristics. Previous studies have shown that Aβ42 features a more rigid and hydrophobic C-terminal region and has a greater propensity to adopt β-sheet conformations than Aβ40 [33]. We hypothesize that these structural differences, particularly in the secondary structure of Aβ42, may hinder its permeation through the polar environment of the monolith and reduce its accessibility to the aptamers immobilized on the mesoporous surface. It is important to note that this is a preliminary hypothesis aimed at rationalizing the observed differences in extraction recovery between Aβ40 and Aβ42. A complete elucidation of the underlying extraction mechanisms and molecular interactions would require further investigation using advanced spectroscopic techniques, which lie beyond the scope of this study.

Indeed, mOS2, is not able to trap Aβ42. Neither mOS1 nor mOS2 was able to extract the Aβ38 peptide. As both isoforms Aβ40 and Aβ42 are central in the pathogenesis of Alzheimer’s disease prognostic [34], we focus on mOS1 for further optimization of the extraction procedure.

**Fig. 4:**
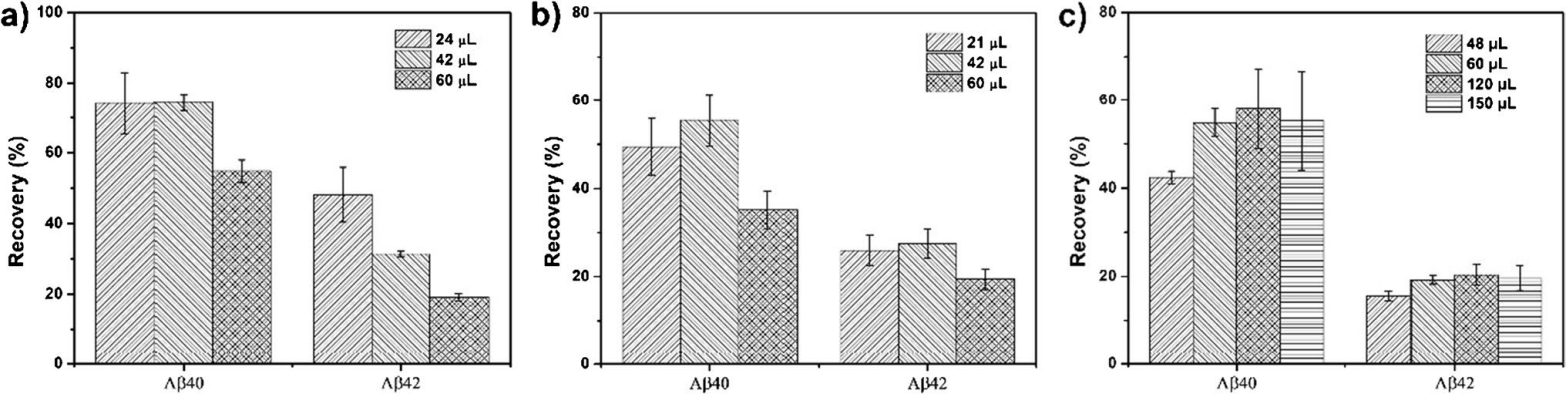
Extraction recovery in the elution fraction after application of the following extraction procedure on the mOS1 monolithic capillary: percolation 60 µL of BB spiked at 1000 ng.mL^−1^followed by (**a)** a washing with different volume of BB and elution with 60 µL of water/acetonitrile 70:30 (*v/v)*, (**b)** a washing with 24 µL of BB, a washing with different volume of water and elution with 60 µL of water/acetonitrile 70:30 (*v/v*), and (**c)** a washing with 24 µL of BB, a washing with 42 µL water and elution with different volumes of water/acetonitrile 70:30 (*v/v*)

After percolating the sample through the mOS (60 µL of spiked BB), the volume of the washing step was evaluated as a function of V_0_ of the capillary. The volumes of the second washing step were maintained constant. The first washing procedure with BB must then be optimized to maintain in the one hand a high retention of analytes and on the other hand to eliminate as much as possible non-specific interactions that can take place on the mOS when applying real samples. Figure 4a shows that increasing the volume of BB (from 1 to 2 dead volume of the mOS), used as the first washing solution, causes better reproducibility as evidenced by the lowest standard deviation observed for the two targeted analytes after washing with 24 and 42 µL of BB. But in the same time, washing step with 60 µL strongly decreased the recovery rate of the two targeted analytes (from 74 to 54% for Aβ40 and from 31 to 19% for Aβ42). The washing step with 24 µL of BB guaranteed the highest recovery rates for Aβ40 and Aβ42 and relative standard deviations lower than 10% it was thus kept for the rest of the study.

However, during the ESI-LC/MS analysis of the elution fraction issued of theses extraction procedure, it was observed that residues of the BB in the elution solution caused significant ion suppression of the targeted analytes (data not shown). Therefore, an additional step of washing with pure water was inserted in the extraction procedure after the first washing step with 24 µL of BB in order to eliminate potential residues of salts before elution of the targeted analytes with 60 µL. Because BB contains higher concentrations of salts, when the elution solution was injected, the signal of the analytes was compromised drastically (data not shown here). Therefore, this additional clen up step using pure water was necessary. As shown in Fig. 4b, a variation in water volume causes a variation in the analyte's recoveries. This variation is probably associated with the influence of the salt contents remaining in the elution solution, which influenced the ESI analytes ionization. Therefore, 42 µL, corresponding to 2 times the dead volume of mOS monolithic capillary, was the volume of pure water required to eliminate the salt residues in the elution fraction. Using 60 µL of water causes a significant loss of analytes by early elution during the washing step.

Finally, the elution step was evaluated. Figure 4c shows that increasing the volume of the elution solution composed of water/acetonitrile 70:30 *v/v* increased the recovery rates of the two targeted analytes. The results showed that 60 µL, 3 times the dead volume, was the minimal volume with minimal RSD values for the quantitative elution of the analytes. Because the viscosity of the elution solution was lower than that of the sample solution, we could increase the elution flow rate to minimize the duration of this step.

Using the optimized condition, the recovery of Aβ40 and Aβ42 in pure media were 74 ± 8% and 48 ± 7%, respectively.

### Capillary binding capacity

**Fig. 5:**
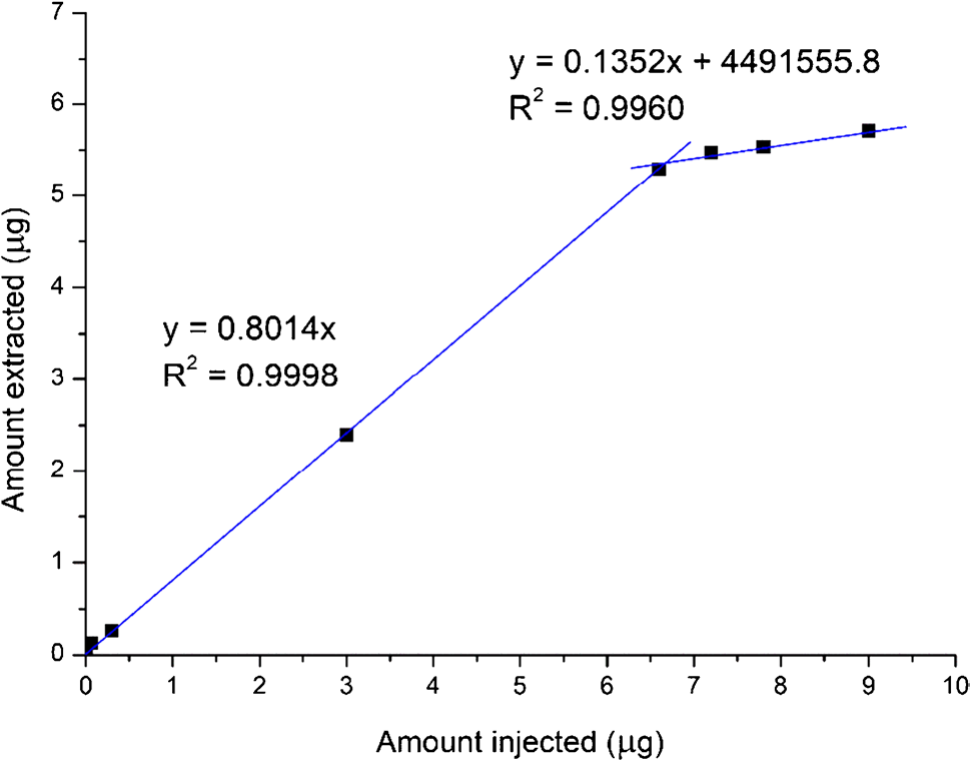
Amount of Aβ40 recovered in the elution fraction versus Aβ40 percolated on the mOS1. The extraction procedure was the same as that described in Fig. 4c

Capacity measurements were thus carried out to evaluate the amount of active immobilized aptamers and to estimate if the extraction procedure optimized will be applicable for the targeted application. The maximum capacity corresponds to the maximum amount of Aβ peptides that can be retained by the mOS without its overloading which results in a decrease of the extraction recoveries thus providing a reliable quantification. Because Aβ40 presents higher recovery rate, the binding capacity was carried out using this peptide. The theoretical extraction capacity (62 µg) should nevertheless be achieved in real extraction because there are some risks of some loss of activity of bound aptamer or problems of accessibility for the peptide. Figure 5 presents the amount of Aβ40 peptide extracted as the function of the amount of Aβ40 peptide percolated through the capillary. The capacity corresponds to the quantity percolated for which a break in the slope of the curve has been obtained. The binding capacity was close to 6.5 µg of Aβ40 which corresponds to 50 pmol of apt 1 by µL of mOS1. This value is 9 times lower than the theoretical calculation. However, the theoretical calculation does not consider the effects of steric hindrance. Depending on the region of the mesopore that the aptamer was grafted, access to the active sites of the aptamer is difficult because of the high molecular mass of Aβ peptide. This value is nonetheless more than sufficient since the targeted concentrations in CSF are of a few ng/mL for Aβ 40 and from one-tenth to one ng/mL for Aβ42 [22, 23].

### Selectivity evaluation

To evaluate the selectivity of mOS1, a different aptamer described to target a small and polar molecule namely homocysteine [35], was grafted to a new monolithic capillary (measured permeability 1.70 × 10^–13^ m^2^ and grafting yield 92%). This capillary, named mOS3, retained less than 15% of the Aβ40 peptide until the elution step. This result corroborated with those presented in Fig. 4, in which the second slope represents the saturation of the active aptamers. After saturation, the slope of the curve decreases drastically and the extraction mechanism in this region is mainly due to non-specific interactions. This slope of the curve, 13.5% (Fig. 4), at these points represents the cross-reactivity. Using an ungrafted hybrid-silica monolithic capillary, the recovery rate for Aβ40 peptide was lower than 3%. These results allowed us to conclude that the non-specific interactions (13.5%) result from interactions between Aβ40 peptide and non-specific sites along the structure of the aptamer, and not between the Aβ40 peptide and the structure of the monolith.

In conclusion, these results demonstrated that, among the total amount of Aβ peptide extracted by mOS1 capillary, less than 15% of the peptides were extracted by non-specific interaction.

### Figure of merits of the analytical validation

Having demonstrated that mOS1 can extract Aβ40 and Aβ42 from pure media, the performance of this support was evaluated on CSF samples. Because Aβ peptides are endogenous molecules, it is difficult to have a blank CSF. Moreover, CSF is difficult to obtain because it is collected by lumbar punction, a painful and expensive medical procedure. For this reason, artificial CSF was used to evaluate the method's performance. This CSF was spiked with Aβ peptides and diluted with BB in proportion CSF/BB 1:2 (*v/v*) before extraction to reduce its viscosity. This dilution step also ensures the solution's pH and ionic strength are close to the extraction condition defined in pure media.

Table 2 shows the main analytical validation parameters for the extraction of the target Aβ peptides on the mOS1 following analysis by UHPLC/MS. This method presented linearity over 1 µg.mL^−1^ in the diluted sample. However, considering the concentration level presented in the real sample of human CSF, the linearity of the method was evaluated from LLOQ to only 13 ng.mL^−1^ [36]. The method exhibited suitable precision and accuracy with CV and RSD values ranging from 1.0% to 12.9% and 4.7% to − 11.1%, respectively. The method achieved LLOQ values of 0.1 ng.mL^−1^ for Aβ40 and Aβ42. This was the lowest concentration that could be quantified, with CV values lower than 20% and RSD values between −20% and 20%. The chromatograms of non-spiked artificial CSF sample showed the absence of interfering compounds in the retention times of the analytes (see Fig. S4 in SI). The method developed here shows excellent extraction performance to quantify Aβ40 and Aβ42 in the synthetic CSF samples. Moreover, each mOS could be used for more than 100 extractions without losing efficiency.

**Table 2:** Main results obtained for analytical validation in artificial CSF

Peptide	Spiked level (ng.mL^−1^)	Precision (CV, %)	Accuracy (RSD, %)
Aβ40	0.6	5.9	3.5
	2.5	5.4	−10.8
	8.0	1.0	4.7
	13.0	6.3	−0.2
Aβ42	0.6	4.4	−11.1
	2.5	2.4	−9.1
	8.0	5.5	2.2
	13.0	12.9	−0.3

The method developed in this study was compared with those published ones, Table 3. Among these methods, the micro-elution SPE plate [37, 38], *fiber-in-tube* SPME [8, 39], and immunoprecipitation [40] have attracted attention. The micro-elution SPE plate [37] present high sampling frequency (high-throughput performance), but it requires high implementation and maintenance costs. In terms of performance, both methods presented similar LLOQ values. However, the extraction based on mOS presented higher selectivity compared to the SPE methods, which use a mixed-mode cation exchange sorbent. Moreover, the possibility of reuse the mOS monolithic capillary constitutes an additional advantage. Compared with the *fiber-in-tube* SPME methods [8, 39], mOS monolithic capillary exhibited better selectivity for Aβ peptides and required less sample volume. The immunoprecipitation method uses antibodies and commercial protocol [40]. Although immunoprecipitation achieved the lowest LLOQ values, this procedure requires multiple sample preparation steps and presents high costs per analysis. Moreover, the antibodies in immunoprecipitation are difficult to reuse.

**Table 3:** Comparison of chromatographic methods to determine Aβ in cerebrospinal fluids

Target analytes	Sample preparation (sorbent)	Matrix (vol., µL)	Chromatographic method	LLOQ (ng.mL^−1^)	Ref
Aβ40 Aβ42	Capillary extraction (aptamer)	CSF (60)	RPLC-MS/MS	0.1	This study
Aβ40	ELISA immunoassay	CSF (50)	Microplate Spectrophotometer	0.7 × 10^–2^	Commercial^a^
Aβ42	ELISA immunoassay	CSF (50)	Microplate Spectrophotometer	1.5 × 10^–2^	Commercial^b^
Aβ38 Aβ40 Aβ42	Micro-elution SPE 96-well plate (MCX)	CSF (200)	RPLC-MS/MS	0.1	[37]
Aβ40 Aβ42	SPE plate (SCX)	CSF (180)	RPLC-MS/MS	0.3 0.4	[38]
Aβ38 Aβ40 Aβ42	Fiber-in-tube SPME (Zw-PIL)	CSF (120)	CapLC-MS/MS	0.1	[39]
Aβ38 Aβ40 Aβ42	Fiber-in-tube SPME (Zw-PIL)	CSF (300)	RPLC-MS/MS	0.4 0.3 0.3	[8]
Aβ40 Aβ42	DSPE (magnetic beads/anti-Aβ antibodies)	CSF (250)	RPLC-MS/MS	0.2 × 10^–1^ 0.1 × 10^–1^	[40]

CapLC: capillary liquid chromatography; DSPE: dispersive solid phase extraction; LLOQ: lower limit of quantification; MCX: cation Exchange; SPE: solid phase extraction; Zw-PIL: zwitterionic polymeric ionic liquid

^a^Invitrogen Human Amyloid beta 40 ELISA Kit (Cat # KHB3481); ^b^ Invitrogen Human Amyloid beta 42 ELISA Kit (Cat # KHB3441)

The mOS demonstrated a huge potential in determining Aβ peptides in real samples and an important advance in the determination of Aβ peptides in CSF as biomarkers for AD.

## Conclusion

A new aptamer hybrid organic-silica monolithic capillaries were developed. Optimization of the synthesis of the hybrid organic-silica monolithic capillary, which exhibited guarantees a monolithic sorbent with adequate stability to reuse in multiple extractions with good repeatability (CV values ranging from 1.0% to 12.9%). Two aptamers selected for their affinity towards Aβ40 peptide were immobilized on the monolithic capillary with very high grafting yields. The preparation of the capillary was very reproducible (n = 3, RSD = 2.9%).

The performance of the aptamer hybrid organic-silica monolithic capillaries as selective extraction device successfully evaluated. Indeed, one of the miniaturized oligosorbent was able to trap selectivity Aβ40 and Aβ42 peptides, whose monitoring in CSF is relevant for the prognostic of Alzheimer’s Disease.

The analytical validation parameters evaluated in artificial CSF were in accordance with the international guidelines and confirmed the performance of the developed method to determine Aβ40 and Aβ42 peptides in this biological fluid.

Our group's future studies will consist of evaluating this aptamer hybrid organic-silica monolithic capillary in online mode. The advantages that we hope to achieve with the online system include increasing the sensitivity of the analyte detection since all portions of the analytes eluted are transferred to the chromatographic column, automating the extraction steps, reducing the exposition of the analyst to solvents, and increasing the analysis reproducibility.

## Supplementary Information

Below is the link to the electronic supplementary material.

Supplementary Material 1 (DOCX 477 KB)

## Data Availability

The authors confirm that the data supporting the findings of this study are available within the article and its supplementary materials. Additional data will be available upon reasonable request.
