# Supplementary material for: Synthesis and characterization of miniaturized aptamer-based monolithic sorbent for selective extraction of β-amyloid peptides from cerebrospinal fluid
Source: Anal Bioanal Chem. 2025 Sep 1;417(26):5907–18. doi: 10.1007/s00216-025-06085-7 (PMC12532768; doi:10.1007/s00216-025-06085-7)
Supplement: Supplementary file 1 — Supplementary Material 1 (DOCX 477 KB) [file 216_2025_6085_MOESM1_ESM.docx]

**Supplementary information**

# Synthesis and characterization of miniaturized aptamer-based monolithic sorbent for selective extraction of β-amyloid peptides from cerebrospinal fluid

Israel Donizeti de Souza^1,2^, Caroline Fernandes Grecco^1,2^**,** Maria Eugênia Costa Queiroz^2^, Valerie Pichon^1^, and Audrey Combès^1*^

^1^ Department of Analytical, Bioanalytical Sciences and Miniaturization (LSABM), Chemistry, Biology and Innovation (CBI), UMR 8231 ESPCI Paris - CNRS, ESPCI Paris, PSL University, 10 Rue Vauquelin, 75005, Paris, France

^2^ Departamento de Química da Faculdade de Filosofia, Ciências e Letras de Ribeirão Preto (DQ-FFCLRP), Universidade de São Paulo, Ribeirão Preto, São Paulo, Brazil.

*Corresponding author: Combès Audrey, E-mail: audrey.combes@espci.fr. Telephone number: ++33140797673

**S1. Quantification by HPLC-UV of the amount of aptamers grafted on the monolithic capillary**

The calculation of the amount of aptamers grafted on the monolithic capillary was performed by quantifying the remaining aptamers in the immobilization and washing solutions. Therefore, 3 solutions were collected during the immobilization steps:

Solution A: 120 µL of the grafting solution + 168 µL 100 mM phosphate buffer solution pH 8.0

Solution B: 720 µL of BB solution containing NaCNBH_3_ at 5 mg.mL^-1^ + 720 µL of BB

Solution C: 420 µL water/methanol 70:30 (v/v) solution

Solutions A, B, and C were dried in N_2_ flow and the dry extract was resuspended in MilliQ water. Then, 10 µL was injected into HPLC-UV system. The chromatographic equipment consisted of an Agilent 1200 series (Agilent Technology, Massy, France) LC system equipped with a binary pump, an autosampler, and a diode array detector controlled by Chemstation software. Ion pairing chromatography was achieved using a

Zorbax Eclipse XDB-C18 column (150 × 3 mm, 5 µm). The mobile phase was composed of 50 mM TEA in water (A) and acetonitrile (B). Chromatographic separation was carried out at 0.2 mL.min-^1^ as follows: 10% B (0 – 2.5 min), 10 – 35% B (2.5 – 10 min), 35% B (10 – 12 min), 35 – 40% B (12 – 13 min), 40 – 10% B (13 – 35 min). Analytes were monitored at 260 nm.

Calibration curves were plotted from 0.5 to 10 µg.mL^-1^ individually for OS1 and OS2, Fig S1. These calibration curves were applied to measure the amount of aptamers in solutions A, B, and C. Solution A corresponds to the amount of aptamers that remain in the effluent of the capillary after the grafting step. Solutions B and C percolated through the capillary to stabilize the grafting process and washed out the aptamers that were not covalently bound to the monolith. Then, the amount of grafted aptamers (*G*) was calculated by the following equation:

$$G=A_{0}-(a+b+c)$$

Where *A_0_* and *a* correspond to the amount of aptamers in the Solution A before and after percolation through the capillary (grafting step), respectively. The *b* and *c* are the amount of aptamers measured in the solutions B and C, respectively







**Figure S1.**Calibration curves for quantification of non grafted (a) apt1 and (b) apt 2.

**
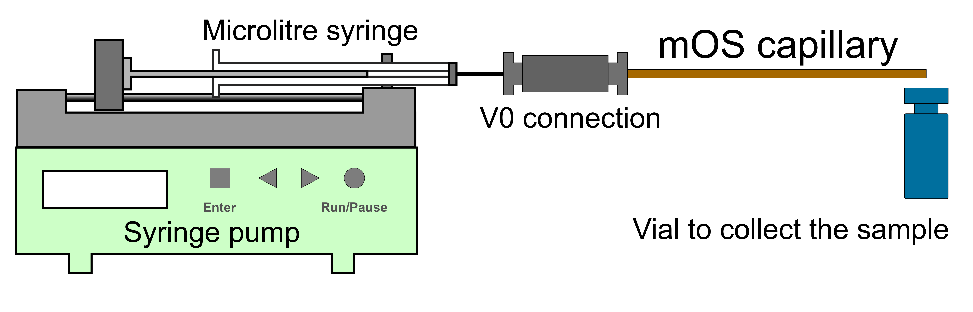
**

**Figure S2.**Experimental set-up for the capillary extraction (*offline*).


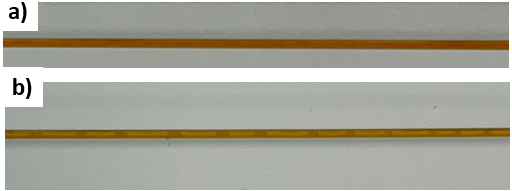


**Figure S3.**Optical images of CapS1 and CapS2 monolithic capillaries





**Figure S4.**Chromatograms of blank CSF samples monitored at *m/z* 1083.3 for Aβ40 (a) and *m/z* 1129.5 for Aβ42 (b).
